# GPRI Biocommentary: Dr. Asmaa Abdel Sameea Mahmoud

**DOI:** 10.1038/s41390-023-02552-y

**Published:** 2023-03-06

**Authors:** Asmaa Abdel Sameea Mahmoud

**Affiliations:** Department of Pediatrics, Faculty of Medicine, Menoufia University Hospitals, Shebin Elkom, Egypt

How glad I was to be invited for the second time as a pediatric research global investigator. I grew up in Berket El Sabaa, Menoufia Governorate, Egypt. I attended Republic Elementary School as a primary school and the Intermediate school was the preparatory school for girls. I grew up with virtuous, struggling parents who took me and my brothers and sisters to the highest positions. My childhood was happy and blessed with a lot of love from my family. I have received several awards and certificates of honor in sports and scientific competitions.

I love helping the sick, the poor, and the orphans, and I learned that from my parents. So, for these reasons, I decided to enroll in medical school.

I joined the Faculty of Medicine, Menoufia University and specialized in pediatrics, especially hematology and oncology.

The first research was done on fungal infections among oncology patients with febrile neutropenia. I was fortunate to work with Professor Farida Hussein Al-Rashidi and her encouragement helped me to complete another research “Evaluation of Mannose Binding Lectin and Interleukin 8 in pediatric oncology patients with febrile neutropenia.” I would like to thank Professor Fady Mohamed El Gendy, and Professor Ahmed Anwar Khatab for their encouragement, and Professor Ghada El Hendway and Professor Enas Essa for their assistance in completing the laboratory assay of this research.

I completed my researches in Pediatrics with my kind and cooperative colleagues in the Faculty of Medicine, Menoufia University (Assistant Professor Nagwan Yossery Saleh, Assistant Professor Nahla Mohamed Said, and Assistant Professor Faten Younis).

To everyone who reads this short article; love, cooperation, purity of soul, and purity of heart are the basis of all success. I wish everyone who worked with me to progress and prosper in all the fields.

**Figure:**
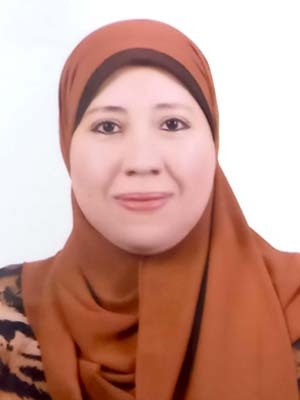
My personal image.

